# A Case of Oral-Vancomycin-Induced Rash in a Patient with Acute Kidney Injury

**DOI:** 10.3390/idr15020019

**Published:** 2023-03-30

**Authors:** Milena Cardozo, Angadbir S. Parmar, Libardo Rueda Prada, Fnu Shweta

**Affiliations:** 1 Mayo Clinic College of Medicine and Science, Rochester, MN 55905, USA; 2 Department of Hospital Medicine, Mayo Clinic Health System, Eau Claire, WI 54703, USA; 3 Department of Hospital Medicine, Mayo Clinic, Jacksonville, FL 32224, USA; 4 Department of Infectious Diseases, Mayo Clinic Health System, Eau Claire, WI 54703, USA

**Keywords:** Vancomycin, drug-related side effects, adverse reactions, oral administration, drug hypersensitivity, pseudomembranous colitis, acute renal failure, and care report

## Abstract

Clostridioides difficile infection (CDI) is one of the most common hospital-acquired infections. Its incidence has increased during the last decade in the community among individuals with no previous risk factors; however, morbidity and mortality are still considered high in elderly patients. Oral Vancomycin and Fidaxomicin are the first lines of treatment for CDI. The systemic bioavailability of oral Vancomycin is thought to be undetectable due to its poor absorption in the gastrointestinal tract; therefore, routine monitoring is not warranted. Only 12 case reports were found in the literature that described adverse reactions associated with oral Vancomycin and its related risk factors. We present a case of a 66-year-old gentleman with severe CDI and acute renal failure who was started on oral Vancomycin upon admission. On day five of treatment, he developed leukocytosis associated with neutrophilia, eosinophilia, and atypical lymphocytes, with no evidence of active infection. Three days later, he developed a pruritic maculopapular rash in more than 50% of his body surface area. Drug Reaction with Eosinophilia and Systemic Symptoms (DRESS) was ruled out since the patient only had three inclusion criteria for this diagnosis. No clear inciting agent was found. Oral Vancomycin was stopped and supportive treatment was supplied for a presumed Vancomycin-induced allergic reaction. The patient had an excellent response, with complete resolution of the rash and leukocytosis in less than 48 h. By reporting this case, we want to raise awareness among clinicians to remember that, albeit rare, oral Vancomycin can be the cause of adverse drug reactions in patients with severe illnesses.

## 1. Introduction

Clostridioides difficile infection (CDI) is one of the most common hospital-acquired infections. The Centers for Disease Control (CDC) estimates that approximately half a million people in the United States of America become infected with Clostridioides difficile each year. Recent studies showed that about 41% of CDI cases are community-acquired [1]. The 2021 CDI treatment guidelines recommend oral Fidaxomicin as the first line of treatment, with oral Vancomycin as an acceptable alternative. Oral Vancomycin is still recommended as the first-line therapy for fulminant CDI [1,2,3]. CDI incidence has increased in the last decade in community-dwelling young and elderly patients without risk factors such as antibiotic use, immunosuppression, previous bowel surgery, and recent hospitalizations [4]. Oral Vancomycin’s systemic bioavailability is considered undetectable due to its poor absorption in the gastrointestinal tract; therefore, routine monitoring is not warranted. Few cases reported dermatological manifestations secondary to oral Vancomycin, including maculopapular rash, Vancomycin infusion syndrome, linear IgA bullous dermatosis, and anaphylactic reaction. Limited literature about adverse reactions to oral Vancomycin and its related risk factors is available. Treatment is directed based on the type of allergic drug reaction and severity of clinical presentation.

## 2. Case Presentation

A 66-year-old gentleman with a past medical history of depression, anxiety, posttraumatic stress disorder, prostate cancer (not on treatment), lower extremity deep venous thrombosis on apixaban, and alcohol use disorder presented to the emergency room after being found by his neighbor in the bathtub confused and covered in feces.

The vital signs of note were a heart rate of 97 bpm, respiratory rate of 22 bpm, blood pressure of 102/61 mmHg, temperature of 37 °C, and oxygen saturation of 88% in ambient air. His physical exam revealed a disheveled patient, all covered in feces, lethargic, cachectic, and in acute distress due to generalized body aches. Mucous membranes were dry. Breath sounds were diminished at bases bilaterally without wheezing or rales. Heart sounds were regular without murmurs, and peripheral pulses were intact. His abdominal exam was benign. Extremities were symmetric, with no evidence of bone deformities. His neurological exam was non-focal, and no skin rash was seen.

The initial laboratory workup was significant for white blood cell count 11.8 × 10^9^/L, hemoglobin 16 g/dL, platelet count 181 × 10^9^/L, sodium 161 mmol/L, potassium 5.2 mmol/L, chloride 115 mmol/L, BUN (blood urea nitrogen) 214 mg/dL, creatinine 9.68 mg/dL, total calcium 8.4 mg/dL, magnesium 3.8 mg/dL, phosphorous 8.2 mg/dL, and lactate 4.4 mmol/L. High anion gap metabolic acidosis with bicarbonate of 13 mmol/L; anion gap 33; and venous blood gas with a pH of 7.22, pCO_2_ 37, and lactate of 4.4. Liver transaminases, total bilirubin, and alkaline phosphatase were within normal limits. Albumin 3.4 g/dL. Ammonia 35 mmol/L. Blood alcohol, acetaminophen, and salicylates were undetected. The urine drug screen was negative. Urinalysis showed turbid urine, leukocyte esterase small, pH 5.0, nitrates negative, ketone trace, bilirubin moderate, and a large amount of blood with white blood cells 21–30/hpf and urine red blood cells 2/hpf. Random urine protein 100 mg/dL. Computerized tomography of the head, cervical spine, chest, abdomen, and pelvis were negative for acute abnormalities. Blood cultures from admission remained negative after five days of incubation. EKG showed normal sinus rhythm, right atrial enlargement, and prolonged QTc 508 msec. The stool test for Clostridioides difficile toxin was positive.

The patient was admitted to the critical care unit (CCU); he received 3 L total of 0.9% sodium chloride fluid bolus followed by maintenance IV fluids of 0.45% sodium chloride at 150 mL/h, 300 mEq IV bolus of bicarbonate followed via a bicarbonate drip (150 mEq of bicarbonate in 1 L of free water at 250 mL/h), thiamine, folate, and oral Vancomycin 125 mg every 6 h. On day 2 of admission, he was started on hemodialysis due to persistent encephalopathy, possibly related to uremia, and no signs of recovery in his kidney function. Fortunately, he only needed two dialysis treatments with slow renal function and mentation improvement. Five days after admission, leukocytosis was noted again, increasing to a peak of 18.6 × 10^9^/L in three days. The white blood cell differential showed neutrophilia of 14.58 × 10^9^/L, monocytosis 1.60 × 10^9^/L, eosinophilia 0.62 × 10^9^/L, basophilia 0.12 × 10^9^/L, and atypical lymphocytes <50% in the peripheral blood smear. Relevant laboratory reports, such as the complete blood cell count with differential, kidney function, and relevant electrolytes, from day 1 of admission until the resolution of the maculopapular rash are summarized in Table 1. The patient remained afebrile and did not have signs or symptoms of worsening CDI or other active sources of infection that could have explained his leukocytosis. A chest X-ray showed no acute findings. Repeated blood cultures were negative. His abdomen remained benign and his diarrhea gradually improved after admission. Eight days after admission, he suddenly developed a diffuse pruritic maculopapular rash on his chest, back, abdomen, and upper extremities. No lymphadenopathy or mucosal involvement was noted. The kidney function was close to his baseline and the liver function test results were unremarkable. His current and home medication list and diet were carefully reviewed, with no possible inciting agent found to be a common explanation for his symptoms. Albeit rare, oral-Vancomycin-induced skin rash was suspected, as no other clear culprit existed. He received treatment with steroids, H_2_ receptor blocker agents, and antihistamines. Vancomycin was switched to oral Fidaxomicin. The patient had complete resolution of the skin rash and leukocytosis 48 h after stopping Vancomycin. He completed ten days of treatment with Fidaxomicin and his diarrhea resolved. The patient was discharged to a skilled nursing facility in stable condition to complete rehabilitation.

## 3. Discussion 

Vancomycin is a tricyclic glycopeptide antimicrobial produced by Streptococcus Orientalis. Its antimicrobial effect consists of inhibiting the polymerization of peptidoglycans in the bacterial cell wall, preventing further transpeptidation of the cell wall synthesis, and leading to the leakage of bacterial components and bacterial death [5]. Vancomycin is FDA (Food and Drug Administration) approved to be administered intravenously to treat Gram-positive bacterial infections in the inpatient setting, such as bacteremia and endocarditis secondary to methicillin-resistant Staphylococcus aureus (MRSA), viridians group Streptococci, Enterococcus, and Corynebacterium species. Vancomycin is also commonly used to treat MRSA lower respiratory tract infections, methicillin-resistant Staphylococcus epidermidis (MRSE), amoxicillin-resistant Enterococcus, skin and bone infections, etc. [3]. Oral Vancomycin and Fidaxomicin are the antibiotics of choice to treat Gram-positive anaerobes, such as Clostridioides difficile causing pseudomembranous colitis, Clostridioides-difficile-associated diarrhea, and staphylococcal enterocolitis [2,3]. CDI has consistently been found as the most common healthcare-acquired infection and is linked with an increased length of stay, elderly morbidity, and mortality. For instance, the main risk factors for CDI are a past medical history of CDI; age >65 years old; recent hospitalization; immunosuppression; pharmacologic agents, such as antibiotics, proton pump inhibitors, H-2 receptor antagonists, and steroids; and the presence of co-morbidities, especially inflammatory bowel diseases and chronic kidney disease. However, recent data from Europe and North America suggest that community-acquired CDI is rising in the 21st century [4,6]. Approximately 20–27% of the cases are community-acquired, with an incidence of 20 to 30 per 100,000 population, mainly affecting low-risk groups, such as children, young patients, antibiotics-naïve patients, or those with no recent hospital admissions. Furthermore, community-acquired CDI was associated with lower mortality but is at risk of a poorer prognosis [1,4].

Vancomycin dosage is based on the type and severity of the infection. The intravenous dose depends on the clinical presentation, kidney function, body weight, and serum trough concentrations. Its pharmacokinetics is complex, as the drug has a short first half-life of about 30 to 60 min and an elimination half-life of 6–12 h. Close monitoring of the Vancomycin level and kidney function is recommended, especially for patients with impaired renal function [5]. In contrast, oral Vancomycin is recommended to treat only gastrointestinal infections caused by Clostridioides difficile, pseudomembranous colitis, and Staphylococcal enterocolitis. Enteral bioavailability is considered negligible at under 10%; thus, a routine therapeutic Vancomycin level is not advocated and does not imply dosage adjustment for renal impairment [5]. In the past decades, some cases of drug-related allergic reactions were reported after oral and rectal Vancomycin administration in cases of severe CDI in critically ill patients [7,8]. Risk factors outlined in some studies relate to the chances of the systemic absorption of oral Vancomycin with renal insufficiency, severe CDI, a high dose of Vancomycin (>500 mg/day), prolonged therapy >10 days, intensive care unit admission, use of retention enemas, and gastrointestinal tract inflammation [9,10]. While intravenous Vancomycin was associated with various adverse drug reactions, an oral formulation seems much safer. However, systemic absorption does increase the adverse drug reaction one would expect with intravenous Vancomycin. Oral Vancomycin is contraindicated in those patients allergic to intravenous Vancomycin due to the risk of exposure causing anaphylaxis.

The most common hypersensitivity reaction to Vancomycin is a skin rash. Various forms include Vancomycin infusion reaction (VIR), previously known as red man syndrome; linear IgA bullous dermatosis; maculopapular and urticarial eruptions; DRESS; IgE-mediated anaphylaxis; and some rare severe cutaneous reactions, such as Stevens–Johnson syndrome, exfoliative dermatitis, toxic epidermal necrolysis, extensive fixed drug eruption, and leukocytoclastic vasculitis, were all described in association with Vancomycin use. Vancomycin hypersensitivity reactions (HSRs) can be classified as either immediate (anaphylaxis) or non-immediate (linear IgA bullous dermatosis, DRESS, Stevens–Johnson syndrome/toxic epidermal necrolysis). Immediate HSRs are usually IgE-mediated reactions, whereas non-immediate HSRs are usually non-IgE or T-cell mediated [11]. VIR is also an immediate hypersensitivity reaction; however, it is not a true allergy and is extremely difficult to distinguish from IgE-mediated reactions. Although renal functions and diarrhea started to improve, our patient developed a diffuse rash on day 8, likely via a non-IgE or T-cell-mediated mechanism. VIR is commonly seen with the rapid infusion of intravenous Vancomycin and is reported with oral Vancomycin [12,13,14,15]. VIR is characterized by flushing, erythema, and pruritus; affects the upper body, neck, and face; and is attributed to the direct activation of mast cells, resulting in histamine release. Hypotension is sometimes present, but VIR is rarely considered life-threatening. Although immunoglobulin E (IgE)-mediated anaphylaxis can have a similar clinical presentation, it can result in angioedema, leading to cardiorespiratory arrest. If someone has experienced anaphylaxis after receiving IV Vancomycin, oral Vancomycin is generally not recommended. DRESS is also known as “drug-induced hypersensitivity syndrome” (DIHS) and “drug-induced delayed multiorgan hypersensitivity syndrome” (DIDMOHS), which is an idiosyncratic skin rash that is an uncommon and potentially life-threatening adverse reaction that was associated with high-risk drugs, such as antibiotics (e.g., Vancomycin, sulfonamide, and antituberculosis agents), anticonvulsants, and allopurinol. The European Registry of Severe Cutaneous Adverse Reactions score (RegiSCAR) and The Japanese Consensus Group criteria (J-SCAR) are the most widely used tools to diagnose DRESS. RegiSCAR requires a minimum of three main criteria: fever > 38 °C, acute rash, lymphadenopathy in at least two sites, and involvement of an internal organ. J-SCAR demands at least five criteria to qualify for atypical DRESS and herpes virus type 6 activation [16]. Linear IgA bullous dermatosis is an autoimmune, vesiculobullous, subepidermal disease characterized by lesions, such as bullous pemphigoid, cicatricial pemphigoid, or dermatitis herpetiformis. Clinical presentation can be observed from days 1 to 14 after medication exposure. Linear IgA bullous dermatosis diagnosis is made via direct immunofluorescence [17].

A PubMed database literature search of case reports and case series for articles published in the English language of dermatologic manifestations associated with oral Vancomycin using the keywords Vancomycin, skin rash, adverse drug reaction, drug hypersensitivity, case report, and oral administration revealed twelve cases [12,13,14,15,18,19,20,21,22,23,24,25], which are summarized in Table 2. Those cases’ severities varied from moderate to severe, with females predominantly affected and 50% of the patients had renal impairment, matching the presentation of our case. Maculopapular rash urticaria was the most common reaction, with 46.1% of the cases, followed by VIR at 30.80% and linear IgA bullous dermatosis at 15.40%. Lastly, one case report described an anaphylactic reaction followed by the first dose of enteral Vancomycin. The time of onset of symptoms documented in Table 2 ranged widely from 1 to 14 days.

In this case, our patient’s risk factors that could have potentially increased the risk of systemic absorption of oral Vancomycin, leading to a diffuse maculopapular rash, were severe acute kidney injury, severe CDI, and CCU admission. The Vancomycin blood levels were not checked in our case. DRESS was excluded after applying the RegiSCAR score; our patient was not febrile and did not have mucosal involvement, lymphadenopathy, or other organ dysfunction. Nevertheless, he had eosinophilia but it was not high enough to meet the RegiSCAR criteria, atypical lymphocytes, and a skin rash extending over more than 50% of the body surface area.

The mainstay of treatment for an adverse drug reaction is to withdraw the offending agent based on a high index of suspicion, which in our case was Vancomycin. Fidoximicin was used to complete ten days of treatment as an alternative line of treatment for CDI. Allergic drug reactions can reasonably be treated with antihistamines in patients with no other systemic symptoms, with resolution expected in the next few days. Patients with VIR secondary to intravenous Vancomycin can be premedicated with an antihistamine and the infusion rate reduced to avoid symptoms; hence, treatment has not been described for oral Vancomycin. Dermatological allergic reactions should be treated based on the disease’s etiology, signs, symptoms, and severity since treatment could differ from others, and no standard of treatment has been established.

## 4. Conclusions

Oral Vancomycin is now commonly used in clinical practice and is often thought to be devoid of systemic adverse reactions because of its negligible oral bioavailability. Some studies favored the systemic absorption of oral Vancomycin under certain risk factors, even though it was considered clinically insignificant. By reporting this case, we want to increase clinicians’ awareness of the possibility of systemic absorption of enteral Vancomycin and its adverse effects, such as skin reactions like those after intravenous administration. Factors that should alert the clinician to suspect systemic absorption of enteral Vancomycin are severe CDI needing hospital admission, especially to the CCU; the Vancomycin dose; the level of renal impairment; and the extent of colonic inflammation. Although most of the skin reactions reported were moderate to severe, there were no case reports that described rare severe cutaneous reactions, such as Stevens–Johnson syndrome, when using oral Vancomycin. Routine serum testing of Vancomycin levels is not recommended.

## Figures and Tables

**Table 1:** Summary of the relevant laboratory blood workup from the time of patient admission to the time of skin rash resolution.

Timeline/ Laboratory Workup	Time of Presentation—Day 1	Day 2	Day 5	Time of Skin Rash—Day 8	Time of Skin Rash Resolution—Day 10
White blood cell count (×10^9^/L)	11.8	7.4	18.6	20.2	10.6
Neutrophils (%)	-	-	14.58	18.19	7.22
Lymphocytes (%)	-	-	1.70	1.20	1.65
Eosinophils (%)	-	-	0.62	0.03	0.33
Hemoglobin (g/dL)	16	15.2	14.5	14	13.8
Platelet count (×10^9^/L)	181	131	256	395	431
Sodium (mmol/L)	161	155	142	135	137
Potassium (mmol/L)	5.2	4.1	3.8	4.7	4.3
Bicarbonate (mmol/L)	13	23	30	27	27
Creatinine (mg/dL)	9.68	9.92	2.27	1.46	1.18
BUN (mg/dL)	214	203	27	29	21
Creatine kinase (U/L)	501	5144	157	-	-

**Table 2:** Summary of the characteristics of published cases of oral Vancomycin allergic reactions.

Case Author	Country	Year of Publication	Age (Years)/Sex	Dose	Adverse Event	Severity	Onset of Symptoms	Renal Impairment
McCullough et al. [18]	USA	1991	82/F	250 mg	Maculo-papular rash	Unknown	8th day	Yes
Killian et al. [12]	Canada	1991	67/F	500 mg	Red man syndrome	Unknown	After first dose	Not reported
Ryosuke et al. [19]	USA	2008	73/F	250 mg	Maculo-papular rash	Unknown	2nd dose after Vancomycin desensitization	Not reported
Bailey et al. [13]	UK	2008	82/F	250 mg	Red man syndrome	Unknown	4th day	Yes
Nallasivan et al. [14]	UK	2009	58/F	Not reported	Red man syndrome	Moderate	3rd day	Yes
O’Brien et al. [20]	USA	2011	45/M	Not reported	Linear IgA bullous dermatosis	Moderate	2nd day of treatment	Yes
Bossé et al. [21]	Canada	2012	35/F	500 mg	Anaphylaxis	Severe	1st dose	No
Choudhry et al. [22]	USA	2015	60/F	Not reported	Linear IgA bullous dermatosis	Moderate	14th day	Not reported
Mizumura et al. [23]	Japan	2015	76/M	500 mg	Maculo-papular rash	Moderate	9th day	No
Baumgartn-er et al. [24]	USA	2017	51/M	250 mg	Maculo-papular rash	Moderate	3rd day	No
Barron et al. [25]	Israel	2018	66/F	Not reported	Maculo-papular rash	Moderate	4th day	No
Arroyo-Mercado et al. [15]	USA	2019	75/F	250 mg	Red man syndrome	Moderate	2nd day	No

## Data Availability

The data presented in this study are available on request from the corresponding author.
